# Diabetes, Obesity, and Endometrial Cancer: A Review

**DOI:** 10.3390/curroncol32120672

**Published:** 2025-11-29

**Authors:** Olivia Hooks, Vama Jhumkhawala, Kristen Sibson, Abbigail Shrontz, Syamala Soumya Krishnan, Sarfraz Ahmad

**Affiliations:** 1 Charles E. Schmidt College of Medicine, Florida Atlantic University, Boca Raton, FL 33431, USA; 2 Department of Medicine, Division of Pulmonary and Critical Care, Downstate Health Sciences University, State University of New York, Brooklyn, NY 11203, USA; 3 AdventHealth Cancer Institute, Gynecologic Oncology Program, Orlando, FL 32804, USA

**Keywords:** diabetes, obesity, endometrial cancer, uterine cancer, comorbid risk factors, metabolism

## Abstract

Endometrial cancer is the fourth most diagnosed cancer in U.S. women. Diabetes and obesity are established independent risk factors for EC, but their combined effect is less defined. This review investigates the literature on these comorbidities as risk factors and modifiers of EC. Multiple cohort and case–control investigations have shown an increased relative risk (RR) and odds ratio (OR) when diabetes and obesity coexist. In one prospective cohort, the RR of EC in diabetic women was 1.94 [95% CI, 1.23–3.08], but increased to 6.39 [95% CI, 3.28–12.06] with obesity; with low physical activity added, RR rose to 9.61 [95% CI, 4.66–19.83]. Case–control studies similarly show an OR of 1.4 [95% CI, 0.9–2.4] for diabetes alone, vs. 5.1 [95% CI, 3.0–8.7] with BMI > 30 and diabetes. Mechanistically, both conditions promote a pro-cancerous microenvironment through metabolic and inflammatory pathways. They also worsen treatment outcomes, with greater surgical complications, thromboembolic events (*p* < 0.01), prolonged hospitalizations 6.2 days versus 4.5 days (*p* < 0.03), and poorer survival with an elevated cancer-specific mortality (HR = 2.65, 95% CI 1.60–4.40). These findings underscore the urgent need for targeted interventions and translational research on how these comorbidities impact the pathophysiologic processes of EC.

## Simple Summary

Endometrial cancer is the fourth most diagnosed cancer in women in the United States. Diabetes and obesity are very common, and both are known to increase the risk of developing endometrial cancer. Yet, their combined effect on the development and outcomes of endometrial cancer is less defined. The purpose of this review is to investigate these relationships. Studies consistently find that the risk of developing endometrial cancer is significantly higher in women with both diabetes and obesity than with each condition independently. Research also suggests that these conditions not only increase the risk of development but also worsen treatment outcomes and long-term survival in endometrial cancer. These findings emphasize the importance of continuing to research how these conditions impact the pathophysiological processes of endometrial cancer and the effectiveness of treatment options, especially with novel therapies.

## 1. Introduction

Uterine corpus cancer, consisting of uterine sarcoma and endometrial cancer (EC), is the fourth most common type of cancer diagnosed in women in the United States (U.S.), behind breast, lung, and colon cancers [1,2]. During her lifetime, a woman in the U.S. has a 1 in 32 chance of developing cancer of the uterus, with the highest risk group being postmenopausal women ages 65–84 years. It is estimated that during this period, a woman has a 1 in 58 chance of being diagnosed with cancer [3]. Globally, uterine corpus cancer in women, with a total of 420,368 new cases and 97,723 deaths reported in 2022 [4]. North America and Eastern Europe had the greatest incidence rates, while Africa had the lowest [5]. Since the mid-2000s, the prevalence rate of uterine corpus cancer has risen by more than 1% each year, with a 0.6% rise in white women and a 2–3% rise in women of all other ethnicities. Entering 2025, it is projected that around 69,120 new cases will occur in the USA for the year [2,6]. This type of cancer is the only kind in the United States to have survival rates worsen in the last four decades [1]. Since 2013 specifically, mortality rates have increased by 1.5% each year, to the current rate of 5.2 deaths in women of all races and 9.5 deaths in non-Hispanic black women per 100,000 cases [2]. In 2025, it is estimated that 13,860 women will die from uterine cancer [6].

### 1.1. Classification of EC

The classification of the EC has evolved throughout the years. In 1983, Bokhman et al. [7] proposed a classification system that incorporated both clinical and epidemiological components. Type I, accounting for 60–70% of cases, consists of estrogen-dependent tumors with endometrial hyperplasia, which are typically present after the age of 50 years and have a better prognosis. Histologically, these tumors generally are endometrioid carcinomas. Type II presents at an earlier age and a worse stage and involves estrogen-independent tumors with endometrial atrophy [8]. While Bokhman’s model is primarily based on clinical and epidemiological features, some notable molecular characteristics exist. Type I tumors are often associated with mutations in the CTNNB1, KRAS, and POLE genes, as well as loss of function in the PTEN tumor suppressor gene and DNA mismatch repair defects. Type II tumors tend to exhibit mutations in TP53, overexpression of HER2/neu, inactivation of p16, and reduction in E-cadherin [9,10].

Microsatellite-unstable (MSI) tumors contain methylation of the MutL homolog 1 (MLH1) gene promoter, resulting in high levels of MSI. The copy number low category comprises microsatellite stable (MSS) cancers, characterized by a lower mutation frequency. The copy number high category comprises MSS cancers with a low mutation frequency but a higher number of copies [8,11]. The four categories include POLE mutated (POLEmut), mismatch repair deficiency/loss mutations (MMRd), no specific molecular profile (NSMP), and p53-abnormal (p53abr) [11,12,13,14]. With these classifications, POLE mutations typically have the best prognosis, while p53 mutations have the worst [14].

However, in 2023, the International Federation of Gynecology and Obstetrics (FIGO) established new guidelines to classify endometrial cancer to better represent tumor biology and prognosis. According to those guidelines, there are histological types that are considered non-aggressive (primarily low-grade endometrioid carcinoma) and aggressive (serous, clear cell, high-grade endometrioid, carcinosarcoma, undifferentiated, mixed, and other rare types). Tumor grade is primarily based on architectural features via biopsy/curettage or hysterectomy specimen and delineated into low- and high-grade. Stages and substages are further defined based on myometrial or ovarian involvement, as well as cervical stromal or lymphovascular space invasion (LVSI). Additionally, the guideline recommends comprehensive molecular classification (POLEmut, MMRd, NSMP, p53) for all endometrial cancers and records this as “m” (e.g., IAm_POLEmut_), indicating that the molecular subtype is known. Defining the histological and molecular subtypes and tumor pattern with FIGO staging criteria, one can better understand the behavior and prognosis of an individual’s endometrial cancer [14].

### 1.2. Pathophysiology of EC

Following the traditional Bokhman classification system, many risk features have been known for Type I EC, which accounts for approximately 70% of all endometrial cancer cases. This cancer is considered estrogen-dependent; exposure of unopposed estrogen on the endometrium leads to hyperplasia and increased risk of malignancy. Endogenous causes of unopposed estrogen include anovulation, polycystic ovary syndrome (PCOS), obesity, early age at menarche, late age of menopause, and nulliparity [15]. Other risk factors include age over 50, family history, hypertension, tamoxifen use, and thyroid disease [15]. Several genetic conditions increase the risk of developing EC. Women with Lynch syndrome are at risk for developing extracolonic malignancies, with the most likely of which being EC [16]. The risk varies depending on the individual gene mutations: PMS2 mutations with a 17% risk, MLH1 and MSH2 mutations with a 54% risk [17], and MSH6 mutations with a 71% risk [17]. Another genetic predisposition, Cowden Syndrome, consists of germline mutations in the PTEN tumor suppressor gene and is estimated to have a lifetime risk of 28% for developing EC [18], compared to the general population’s risk of around 3% [2].

### 1.3. Diagnosis of EC

EC typically presents as abnormal uterine bleeding or postmenopausal bleeding. In particular, vaginal bleeding is the initial sign in 90% of EC cases in postmenopausal women [19]. The workup for EC involves a comprehensive medical history and physical exam, risk factor assessment, laboratory tests and imaging, and additional considerations for pre- vs. postmenopausal women [15]. In premenopausal women, ultrasound measurement of endometrial thickness is less reliable and of no diagnostic value due to fluctuations in endometrial lining thickness throughout the menstrual cycle [15]. Therefore, the American College of Obstetricians and Gynecologists (ACOG) recommends against identifying or utilizing this measurement [15]. However, an endometrial biopsy may be considered based on clinical symptoms and physician judgment [15].

In postmenopausal women, vaginal bleeding always requires further workup to rule out malignancy, which typically includes a transvaginal ultrasound (TVUS) with or without a histological analysis of the endometrium. The imaging aims to determine the thickness of the endometrial lining [15,19]. In postmenopausal women, a threshold of four mm for the endometrial thickness provides a negative predictive value (NPV) of 99.3% and a sensitivity of 96.2% for EC according to an extensive systematic review of 44 studies [20,21]. If the lining is four millimeters or thicker and the patient presents with vaginal bleeding, the clinician should pursue additional testing with either an endometrial biopsy, office hysteroscopy, or sonohysterography [20]. Of note, an incidental finding on TVUS of endometrial thickness greater than 4 mm does not always warrant further evaluation; however, if risk factors such as PCOS, obesity, type 2 diabetes mellitus (T2DM), or the use of unopposed estrogen are present, additional workup may be necessary [19]. The gold standard for diagnosis is a histological analysis obtained from an endometrial biopsy via outpatient sampling with disposable devices or hysteroscopy-guided endometrial biopsy [15,19,20,21]. Sampling with disposable devices is reliable and accurate, but if a surgical approach is favored or bleeding persists despite a normal biopsy, the clinician may pursue a hysteroscopy and D&C [15,19,20,21]. Although hysteroscopy is not required, ACOG recommends its use to evaluate discrete lesions or the background endometrium [15].

Understanding the complexity of EC and the many associated risk factors, this review aims to comprehensively examine the current peer-reviewed literature on diabetes and obesity as major comorbid risk factors and disease modifiers for EC. It was hypothesized for this review that if patients have diabetes and obesity, then the risk of developing and worsening outcomes for EC is greater than if patients have either factor independently.

## 2. Overview of Diabetes in EC

Diabetes has emerged as a major concern globally, with its prevalence projected to continue to rise significantly. In 2017, an estimated 451 million adults were living with diabetes, representing a 58% increase from 2010, with the most significant rise occurring in the elderly [22]. Estimates suggest that nearly half of the individuals with diabetes remain undiagnosed [22].

In the United States, diabetes has seen a more than five-fold increase in prevalence from 1975 to 2010 [23]. As of 2024, 11.6% of the U.S. population—approximately 38.4 million people—have diabetes [23]. The percentage is even higher among older adults, with about 30% of individuals over age 65 years affected, with the incidence of cases reaching approximately 1.2 million Americans every year [23]. While gender differences in the prevalence are not pronounced, diabetes rates are nearly twice as high among Hispanic and African American populations [23]. Additionally, lifetime risk estimates suggest that 32.8% of men and 38.5% of women in the U.S. will develop diabetes [23]. Those diagnosed at age 40 years have an estimated 11-year reduction in lifespan for men and 14 years for women [24].

Diabetes remains a leading cause of mortality worldwide. The American Diabetes Association (ADA) identified diabetes as the eighth leading cause of death in 2021. However, mortality estimates are likely underreported since diabetes often contributes to life-threatening events such as heart attacks and strokes, but is not always listed as the primary cause of death on certificates. It is estimated that diabetes-related mortality ranges from 6% to 10% globally, with approximately 73% of diabetes-related deaths occurring before the age of 60 years [22]. In the U.S., diabetes-related mortality was estimated at 3.1% in one study, though this is likely an underestimate [24].

Among the many complications associated with diabetes, its link to cancer, particularly EC, has drawn increasing attention. Although the absolute incidence of EC among diabetic women is relatively low, given the widespread prevalence of diabetes, studies suggest a significantly increased risk. Some research estimates that diabetic women have a standardized incidence ratio for EC as high as 1.69 compared to the general population [25]. It is estimated that 10% to 20% of women diagnosed with EC also have diabetes, though this proportion varies based on population characteristics [25]. The presence of diabetes EC in patients has significant prognostic implications. Numerous investigations have studied the link between EC and diabetes. For instance, one study indicates that the five-year OS rate in diabetes EC patients is lower than for those without diabetes, with a hazard ratio (HR) of 1.4 (CI 0.9–2.2) [26]. However, there is no significant link between EC and diabetes -specific mortality.

**Figure 1:**
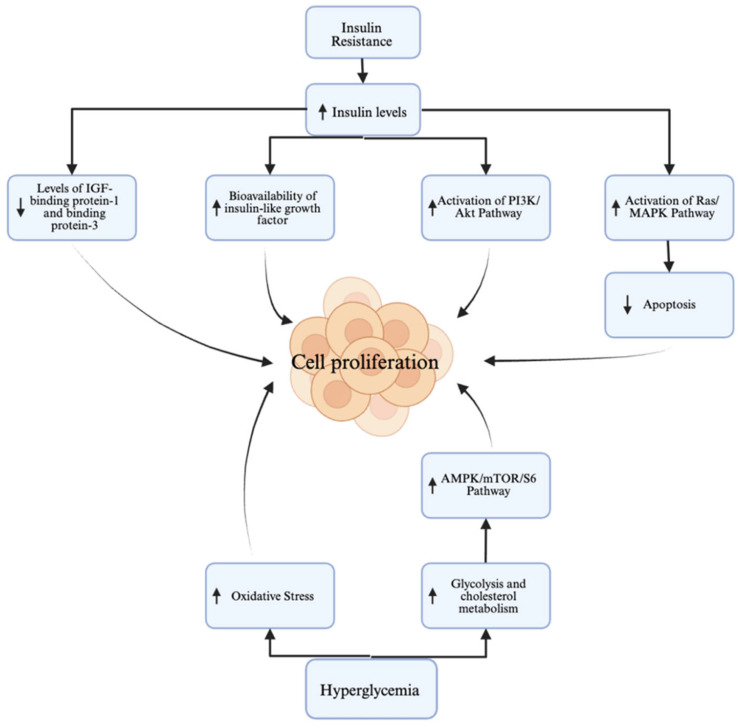
Proposed mechanisms of hyperglycemia and insulin resistance linking Type II Diabetes Mellitus to cell proliferation as seen in endometrial cancer. Created in Biorender. Vama Jhumkhawala. (2025). https://BioRender.com.

An in vitro study has shown that chronic hyperinsulinemia, a hallmark of type 2 diabetes, promotes EC development through direct and indirect mechanisms. In this study, insulin stimulates cell proliferation via the survival pathways (PI3K/Akt and MAPK) [27]. However, in vivo studies have shown that insulin resistance leads to elevated levels of circulating insulin, which increases the bioavailability of IGF-1, further promoting cellular proliferation and survival. The insulin and IGF axis enhances cellular growth, contributing to an increased cancer risk due to anti-apoptotic effects, as seen in Figure 1 [27].

Hyperglycemia also plays a role in EC pathogenesis, as high glucose levels contribute to EC cells’ growth and metastasis. Hyperglycemia enhances glycolysis, which is critical for cancer cell metabolism and proliferation. This metabolic shift is mediated by pathways such as AMPK/mTOR/S6 and MAPK, which are upregulated in a high-glucose environment [28]. This mechanism, in tandem with the effects of insulin resistance, can be seen in Figure 1. Additionally, diabetes is linked with chronic low-grade inflammation and elevated oxidative stress, both of which are implicated in carcinogenesis.

Emerging evidence suggests that miRNAs may serve as molecular links between insulin resistance and EC [29]. Dysregulation of specific miRNAs involved in insulin signaling and glucose metabolism has been associated with both conditions, highlighting their potential role in the pathogenesis of EC in diabetic patients [29]. Thus, the pathophysiology of diabetes as a risk factor for EC involves a complex interplay of hyperinsulinemia, hyperglycemia, chronic inflammation, oxidative stress, hormonal changes, and miRNA dysregulation. These mechanisms collectively contribute to increased endometrial cell proliferation, reduced apoptosis, and heightened risk of cancer. Furthermore, while another study assessed the role of obesity-related miRNA changes, the connections do suggest a possible overlap with pathways implicated in diabetes and metabolic dysfunction [29].

### 2.1. Diabetes as a Risk Factor

Diabetes is an established risk factor for the EC. Several studies have identified hyperinsulinemia, insulin resistance, chronic inflammation, oxidative stress, and hormonal alterations as significant risk factors for diabetic EC patients [30,31,32,33]. It is important to note that Fader et al. [33] suggested that many biomarkers associated with EC and diabetes are more closely linked to obesity rather than diabetes itself, highlighting the complex interplay between metabolic factors and cancer risk [33]. Additionally, elevated levels of C-peptide have been linked to a higher risk of EC. Lukanova et al. [34] found that increased circulating C-peptide levels were linked with an elevated risk of growing EC, even after adjusting for BMI and other confounding factors.

As noted earlier, the underlying mechanisms linking hyperinsulinemia to EC involve both direct and indirect effects. Insulin acts as a mitogenic factor, promoting cellular proliferation and inhibiting apoptosis by activating the PI3K/Akt and MAPK signaling pathways [35]. However, despite the link between insulin resistance and cancer risk, a study by Morielli et al. [36] found no significant links between insulin resistance or inflammation biomarkers and mortality outcomes in patients with EC, suggesting that other factors may play a more prominent role in disease prognosis.

Patients with diabetes, characterized by elevated fasting insulin levels, higher HOMA-IR values, and increased C-peptide levels, are more likely to develop endometrioid endometrial carcinoma (EEC). Thus, elevated fasting insulin levels, higher HOMA-IR values, and increased C-peptide levels serve as important biomarkers in patients with diabetes that correlate with a higher risk of EC. While obesity-related metabolic dysfunction is a key contributor, studies suggest that diabetes itself exerts independent effects on EC. Experimental and epidemiological findings underscore the role of hyperinsulinemia, hormonal alterations, and inflammatory pathways in the pathogenesis of EC. However, the impact of these mechanisms on cancer progression and mortality remains an area of ongoing research.

### 2.2. Diabetes as a Prognostic Factor

Research into biomarkers that correlate with higher EC risk in diabetic patients is ongoing. However, most biomarkers associated with EC in diabetic patients are more closely linked to obesity than diabetes itself [33]. Recent studies have found no significant link between insulin resistance and inflammation biomarkers, as well as mortality outcomes in EC patients [36]. Although the link between diabetes and EC mortality is weaker, there is evidence suggesting an elevated risk. The mortality risk associated with diabetes in EC patients has been reported to have a relative risk of 1.23 (CI 1.02–1.47) [37]. Han et al. reviewed the etiological links between diabetes and EC, emphasizing that patients with diabetes are twice as likely to develop EC, likely due to hyperglycemia and insulin resistance promoting cancer cell growth and invasiveness [27]. These studies collectively support the conclusion that diabetes mellitus is a significant risk factor for the development of EC.

The impact of diabetes on the treatment and survival of EC patients is multifaceted. Treating diabetes, particularly with metformin, can improve prognosis. Metformin has been shown to significantly reduce overall mortality in patients with EC across various clinical stages compared to those not treated with metformin, which is theorized to promote cell proliferation by decreasing Ki67 proliferation [38]. The impact of diabetes on treatment, survival, and quality of life (QoL) is complex, with metformin treatment being associated with more prolonged RFS in EC patients, while insulin therapy has been linked to the risk of cancer deaths [39].

Diabetes mellitus, particularly type 2 diabetes (T2DM), is a well-established and independent risk factor for EC. Interactions between the processes of hyperinsulinemia, insulin resistance, chronic inflammation, oxidative stress, hormonal dysregulation, and emerging molecular mechanisms drive this relationship [34,35,36]. While obesity contributes significantly as a risk, diabetes exerts its biological effects on endometrial tissue as well. The presence of diabetes not only increases the incidence of EC but also negatively influences prognosis and survival, indicating the need for screening and management in high-risk populations [37]. Given the close link between diabetes and obesity, it is essential to examine the role of obesity more specifically, both as an independent risk factor and as a modifier of the relationship between diabetes and EC development and progression.

## 3. Overview of Obesity in EC

Obesity is a significant public health issue and leads to a variety of chronic conditions and premature mortality [40]. Similar to the rise in the prevalence of obesity, the overall mortality rate due to obesity has steadily increased from 1.8 per 100,000 people in 2010 to 3.1 in 2020 [40]. A notable increase in this mortality rate occurred in 2019 and 2020, during the global pandemic, likely attributable to the rise in cardiovascular disease, a major obesity-associated complication that affects obesity-related health outcomes [40]. The relative risk among non-smokers provides a more valid estimate of the actual effect of elevated BMI on mortality from cancer, as it eliminates the confounding variable of smoking, a known risk factor for cancer and cancer-related deaths [41]. Regarding EC, the link between obesity and carcinogenesis is likely to have a hormonal basis [42]. The development of EC is believed to be attributable to unopposed estrogen levels stimulating endometrial epithelial cells and proliferation [42]. Aromatization is the process by which androgens, such as androstenedione, are converted to estrogens in adipose tissue by the enzyme aromatase [42]. Therefore, adipose tissue, which is in higher concentration in people with obesity, is a significant extra-gonadal source of estrogens [43]. In pre-menopausal women, obesity may cause luteal phase progesterone deficiency, which causes a relative increase in estrogen levels and can predispose individuals to the development of EC [42].

**Figure 2:**
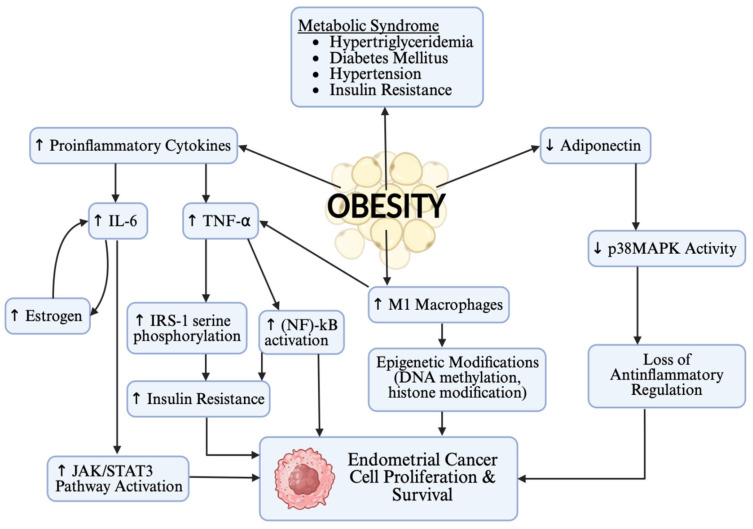
Biochemical pathways linking obesity, chronic inflammation, and endometrial cancer development. Created in Biorender. Kristen Sibson. (2025). https://BioRender.com.

TNF-α encourages tumor development through multiple pathways. M1 macrophages release TNF-α, which activates the nuclear factor (NF)-κB signaling pathway, thereby inhibiting cancer cell apoptosis [44]. Furthermore, TNF-α induces serine phosphorylation of insulin receptor substrate-1 (IRS-1), inhibiting the activation of downstream signals, leading to insulin resistance and contributing to EC development [44]. Figure 2 illustrates the complex interactions between obesity-related cytokines and signaling pathways that regulate EC cell proliferation and survival.

Adiponectin is a biomarker that is inversely associated with EC, and lower levels are often found in obesity due to concurrent hyperinsulinemia and a degree of insulin resistance [33]. Through activation of the downstream LKB1-AMPK/S6 signal axis, adiponectin can inhibit EC cells’ proliferation, adhesion, and invasiveness [44]. Adiponectin can also enhance the insulin sensitivity of EC cells through the AMPL/S6K1/IRS1 signaling pathway and activation of p38MAPK activity [44]. Furthermore, adiponectin can alter the tumor immune microenvironment through the p38MAPK signaling pathway, promoting the transformation of M2 tumor-associated macrophages to the M1 type, inhibiting the growth of tumors [44].

The activity of vascular endothelial growth factor-mammalian target of rapamycin (VEGF-mTOR) in obese EC patients is significantly higher than in non-obese patients [44]. Excess adipose tissue can activate the Akt/mTOR pathway and promote both the proliferation and invasion of cancer cells, suggesting this signaling pathway may play a role in the pathogenesis of EC and serve as a potential therapeutic target [44]. Furthermore, prolactin and thyroid-stimulating hormone (TSH) have various metabolic and physiological effects [33]. Irregular levels of these hormones can cause menstrual dysfunction and impact the development of EC [33]. For screening purposes in high-risk populations, such as obesity, these hormones have the potential to discriminate between healthy individuals and those with EC [33]. A study focused on EC and various biomarkers showed that measuring prolactin levels accurately discriminated between healthy and diseased individuals with 98.3% sensitivity and 98% specificity [33].

### 3.1. Obesity as a Risk Factor

Furthermore, Kalliala et al. [45] published an umbrella review of meta-analyses in 2017, highlighting the relationship between obesity and obstetric and gynecologic conditions, including the development of EC. Two meta-analyses examining the link between obesity and increased risk of EC met criteria for substantial evidence, and nine met criteria for highly suggestive evidence [45]. The link of BMI, per 5-unit increase, with the incidence of EC in pre-menopausal women was supported by substantial evidence, with the most extensive study showing a RR of 1.53 [95% CI, 1.48–1.58], and in postmenopausal women was supported by highly suggestive evidence with a RR of 1.51 [95% CI 1.45–1.58]. The evidence supports the link between BMI and the development of both type I and type II EC.

BMI is a well-established risk factor for endometrial cancer; however, there are potential limitations to using this measurement in studies. BMI reflects both fat and fat-free mass and does not assess the distribution of fat, which can vary significantly even among individuals with a similar BMI [46]. Other measures provide more precise estimates of overall fatness than BMI, including body fat percentage and fat mass, but it is unknown whether these measures are more strongly related to endometrial cancer risk than BMI [46]. A prospective study analyzing over 135,000 postmenopausal women found that although more precise measures of overall adiposity exist, they are not better indicators of endometrial cancer risk compared to BMI [46].

### 3.2. Obesity as a Prognostic Factor

While obesity is a known risk factor for the development of endometrial cancer, it also contributes to the clinicopathologic characteristics of EC and can affect prognosis. A retrospective study of 406 patients diagnosed with EC showed that obesity is correlated with cervical stromal invasion (CSI) in both type I (*p* = 0.022) and type II (*p* = 0.019) EC [47]. Deep CSI, defined as the inner two-thirds vs. the superficial or outer one-third, has been found to be an independent predictor of mortality with a hazard ratio of 2.8 [48]. This type of invasion is associated with increased risk of lymph node metastasis and recurrence of disease [48].

Given that obesity contributes to all-cause death in EC patients, it is vital to evaluate the influence of weight loss on this condition. Weight loss decreased CRP levels by 33.5%, with the most substantial effect observed with a weight loss of >7%, and decreased IL-6 levels by an estimated 41.9% [49]. The authors concluded that greater weight reductions are necessary to decrease TNF-α significantly. However, 10% or greater weight loss resulted in an average decrease in TNF-α by 13% [49]. Circulating leptin levels, which promote inflammation, were lowered with any weight loss percentage [49]. There was no statistically significant reduction in estradiol or testosterone due to weight loss [49]. Naqvi et al. [50] demonstrated a significant increase in CD8+ cells, accompanied by a reduction in weight and BMI (*p* = 0.0097 and *p* = 0.0093, respectively).

A deeper understanding of how weight loss contributes to the course and overall mortality of EC can help guide treatment options. Lifestyle modifications, surgical interventions, and pharmacological approaches have all been shown to significantly reduce body weight. However, they have differing efficacy, with a meta-analysis of 38 studies showing that bariatric surgery, compared to lifestyle or pharmacotherapy interventions, results in a 25.8% greater reduction in weight [49]. Ward et al. [51] published a retrospective cohort study in 2014, which compared the risk of developing uterine malignancy in obese women with and without a history of bariatric surgery for weight loss. When compared to obese women with no history of bariatric surgery, the relative risk of uterine malignancy was 0.19 [95% CI, 0.17–0.22] and 0.48 [95% CI, 0.43–0.55] in admissions of women who, post-bariatric surgery, were classified as normal weight or obese, respectively.

Immune checkpoint inhibitors (ICIs) are becoming part of the first-line treatment for endometrial cancer [52], and as 80% of EC-diagnosed women are obese, it is important to consider the influence of obesity on the response to immune-based therapies [53]. A retrospective study consisting of 524 patients with EC showed that overweight and obese patients had significantly prolonged progression-free survival (PFS) (overweight versus normal BMI: median 6.5 versus 4.5 months, HR 0.71, 95% CI 0.55–0.93, *p* = 0.0112; obese versus normal BMI: median 7.8 versus 4.5 months, HR 0.61, 95% CI 0.47–0.78, *p* < 0.0001) and overall survival (OS) (overweight versus normal BMI: median 27 versus 15.2 months, HR 0.61, 95% CI 0.45–0.83, *p* = 0.0018; obese versus normal BMI: median 22 versus 15.2 months, HR 0.65, 95% CI 0.49–0.86, *p* = 0.0026) following treatment with an ICI, including pembrolizumab, durvalumab, nivolumab, or a combination of ICIs, when compared to patients with normal BMI [53]. Among the 307 patients who received pembrolizumab in combination with lenvatinib, the median PFS was 7.3 and 8.2 months for overweight and obese patients versus 5.6 months for normal-weight patients [HR 0.62, 95% CI, 0.45–0.87; HR 0.57, 95% CI, 0.42–0.79, respectively], and median OS was 27.7 and 21.1 months versus 14 months [HR 0.53, 95% CI, 0.35–0.79; HR 0.64, 95% CI, 0.45–0.92, respectively]. Gómez-Banoy et al. [53] established that these associations persisted after adjusting for EC molecular subtypes and relevant clinical factors. Obesity was also shown to be associated with a higher rate of immune-related adverse events (irAEs), suggesting an enhanced immune response following treatment with ICIs in this population [53].

## 4. Overview of Diabetes and Obesity as Comorbidities in EC

As noted earlier, the literature supports the link between diabetes and obesity as separate risk factors and prognostic indicators of EC; however, less is known about how these two conditions, when co-occurring, affect the development and mortality of EC. Given the available literature, multiple studies have shown an even greater risk of EC when patients have both diabetes and obesity, as seen in Table 1. Friberg et al. [54] conducted a population-based prospective cohort study involving 36,773 women, which resulted in 225 incident EC cases after a seven-year follow-up period with a mean age of 68.6 (+/−9.5) years. The authors then analyzed the link between diabetes and EC, as well as how obesity and physical activity influence this link. When compared to non-diabetic women, the relative risk for developing EC in women with diabetes was 1.94 [95% CI, 1.23–3.08]. When obesity was added as a co-morbidity with diabetes, the relative risk increased to 6.39 [95% CI, 3.28–12.06]. Furthermore, when women who were classified as diabetic, obese, and had low physical activity, the relative risk was the greatest at 9.61 [95% CI, 4.66–19.83] [54]. This demonstrates that having diabetes led to a statistically significant ~2-fold higher risk for developing EC, but having diabetes and obesity had a statistically significant ~6.5-fold increase.

Combined data from two case–control studies by Lucenteforte et al. [29] also looked at diabetes and the risk of EC, with the effect of modification of body weight and physical activity. In a total of 777 women with EC and 1550 control women, both with a median age of 61 years, the OR in cases with diabetes and no obesity was 1.4 [95% CI, 0.9–2.4]. However, when BMI > 30 kg/m^2^ at diagnosis was included with diabetes, the OR increased to 5.1 [95% CI, 3.0–8.7]. Salazar-Martinez et al. [55] reported similar findings in another case–control study examining the risk of EC among Mexican women with diabetes and obesity. The authors compared 85 histologically confirmed EC cases with 668 population-based controls. They found that in women with diabetes and obesity, there was an eight times greater risk of EC (OR = 8.0, CI = 2.8–22.7).

**Figure 3:**
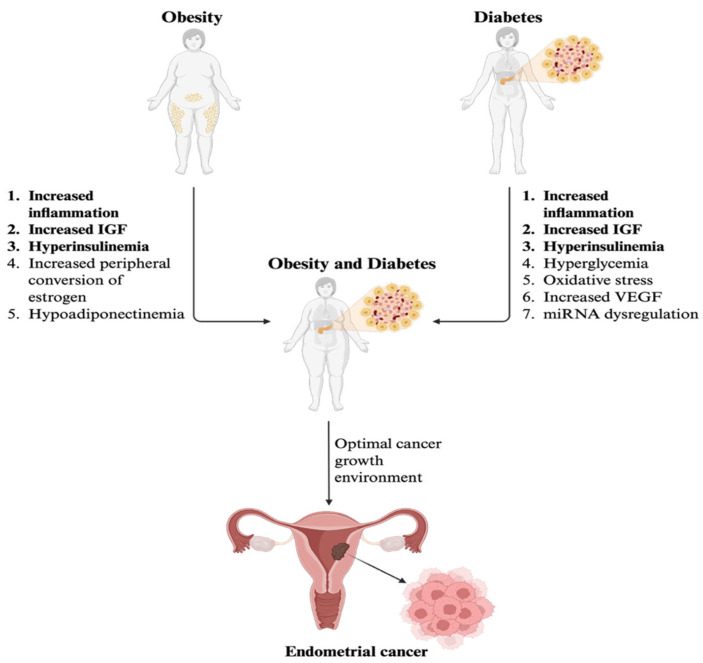
Contributing factors of obesity and diabetes as comorbidities in endometrial cancer development. Created in BioRender. Olivia Hooks. (2025). https://BioRender.com.

Therefore, there seems to be an interplay between diabetes and obesity that makes the risk so much greater when patients have both conditions. The combination of metabolic and inflammatory disturbances creates a microenvironment susceptible to cancer cell growth. While obesity induces a state of inflammation, hypoadiponectinemia, hyperinsulinemia, increased peripheral conversion of estrogen, and increased IGF, diabetes further contributes to the optimal environment for cancer growth by increasing IGF, glucose, and VEGF, as shown in Figure 3. This hyperglycemic, insulin-resistant microenvironment leads to precancerous and cancerous endometrial cell proliferation and tumor progression [54,56].

Diabetes and obesity increase the risk of developing EC, but once a patient with diabetes and obesity has the diagnosis of EC, their co-morbidities further impact their treatment and survival. A cohort study of 1359 Australian women diagnosed with EC found that when patients had both diabetes and obesity, there was an increased HR for cancer-specific mortality (HR = 2.65, 95% CI 1.60–4.40) [57]. There is also an increase in surgical candidacy limitations and post-operation and treatment-related complications associated with patients who have diabetes and obesity [3,58]. Especially in patients who are obese, there is a higher risk for anesthesia-related complications and respiratory distress perioperatively [58]. Bouwman et al. [59] discussed the increase in complications, such as wound infections, and antibiotic use occurred more frequently in open EC surgery, and morbidly obese patients were at the highest risk. A study by Yin et al. [60] looked at post-operative outcomes and incidence of deep vein thromboses (DVT) in 219 patients with EC who were treated between 2002 and 2012. The authors divided them into groups based on their co-morbidities of diabetes, obesity, and/or hypertension. However, there was a significant difference in the length of hospital stay (LOS) among patients with both diabetes and obesity compared to controls, 6.2 days versus 4.5 days, respectively (*p* < 0.03). There was also a statistically significant difference in venous thromboembolic (VTE) events between combined diabetes and obesity patients and controls with incidence of DVT in the combined group accounting for eight of the 15 cases (*p* < 0.01) and of the three cases of pulmonary embolism (PE) documented, two of the three were from the combined group (*p* < 0.01).

Thus, diabetes and obesity as comorbidities share many pathophysiologic mechanisms to account for the higher risk of developing EC. This is clinically important as it stratifies patients into different risk categories and requires healthcare providers to have a lower threshold for suspecting EC, especially when patients present with non-specific symptoms. Furthermore, many studies have shown a statistically significant difference in mortality, partially due to treatment complications and limitations when these two conditions are combined.

**Table 1:** Endometrial cancer, diabetes, and obesity as comorbidities.

References	Primary Findings
Baker-Rand [3]	Co-morbidities increase surgical candidacy limitations and post-operation and treatment-related complications.
Travaglino [13]	Meta-analysis of six studies identified 3132 endometrial cancer cases, a RR of 1.89 for those with obesity, diabetes, and dyslipidemia.
Lucenteforte [29]	Two case–control studies with 777 cases of EC and 1550 controls (OR for diabetes only = 1.4, diabetes + obesity = 5.1)
Friedenreich [32]	In those with obesity, diabetes, and dyslipidemia, an OR of 1.53 was identified for EC.
Friberg [54]	Risk of developing EC: Diabetes alone increases the risk by 2-fold, diabetes combined with obesity by 6.5-fold, and diabetes combined with obesity and physical inactivity by 9.5-fold.
Nagle [57]	Hazard ratio for EC-specific mortality in patients with diabetes and obesity was 2.65 [95% CI 1.60–4.40]
Qiang [58]	Diabetes and obesity led to a higher risk of anesthesia-related complications and respiratory distress perioperatively.
Bouwman [59]	Morbidly obese patients were at the highest risk of wound infections and antibiotic use in open EC surgery.
Yin [60]	Patients with diabetes and obesity had an increased length of hospital stay (6.2 days vs. 4.5 days, *p* < 0.03) and a higher incidence of venous thromboembolic events (*p* < 0.01) compared to the control group.

*Abbreviations*: EC = Endometrial cancer; CI—Confidence interval; OR = Odds ratio.

## 5. Treatment Considerations in EC

Additionally, despite improvements in surgical and adjuvant treatments, the recurrence rate for EC remains around 14–18%, with 78.1% of those recurrences occurring within the first two years post-diagnosis [61,62]. Recurrence is also dependent on molecular subtype, with MMR-d tumors resulting in a more localized pattern of recurrence and the shortest median time to recurrence (16 months, 95% CI 12–20 months), while p53 tumors are more likely to have abdominal recurrences (*p* = 0.042). The MMR-d tumors have the best median survival after recurrence (43 months, 95% CI 11–76) compared to the worst median survival with p53 tumors (10 months, 95% CI 7–13, *p* = 0.001) [62]. Knowing the aggressive nature and death toll that EC has on patients, it is essential to identify novel translational research to better manage and treat this disease.

In general, treatment of EC is based on stage and pathological characteristics of the disease [14]. Undergoing comprehensive surgical staging using the FIGO staging system for EC allows for a greater understanding of disease extent [14]. Surveillance and treatment protocols vary based on histological and molecular risk stratification, but also require adjustments based on patients’ tolerance to treatment, symptoms, and goals [63].

The standard of care for early-stage EC is a total hysterectomy with bilateral salpingectomy (BSO) via minimally invasive surgery (MIS) [15,63]. In addition, evaluation of the lymph nodes via lymphadenectomy or sentinel node biopsy can assess metastatic disease; however, the utility and extent of resection vary among physicians [63]. In patients with uterine-confined disease and only surgical treatment, which accounts for around 55% of EC patients, there is an estimated 95% chance of 5-year RFS [63]. Despite surgery being a mainstay for EC treatment, it may not be an option for all patients. Those who have early-stage disease or are not optimal surgical candidates, like those with diabetes and obesity, or have late-stage or recurrent cancer, may consider hormone therapy. Progestin therapies, like oral progestins or a levonorgestrel intrauterine device (LNG-IUD), are helpful for fertility-sparing, non-surgical options. Around 9% of patients diagnosed with EC are younger than 44 years old, so progestin therapy is vital for premenopausal patients who desire future pregnancies [15,64].

Other options for managing EC include adjuvant treatment with radiotherapy, chemotherapy, and/or immunotherapy, which can also be used in conjunction with surgery [63]. A meta-analysis of eight trials compared adjuvant radiotherapy post-hysterectomy and no adjuvant treatment for stage 1 EC [65]. Chemoradiotherapy for advanced-stage EC, outcomes can be further improved than either therapy independently [15]. For advanced or recurrent EC, multiple studies have reported that chemotherapy with paclitaxel and carboplatin is the best first-line treatment with less toxicity and is as effective as other chemotherapy regimens [15,66,67].

While chemotherapy and radiation have been available for many decades to treat EC, translational research into novel immunotherapies has the potential to bring about groundbreaking changes in improving the survival and management of EC, especially for patients with metastatic or recurrent disease [68]. In 2024, the FDA approved three new therapy regimens with monoclonal antibodies for EC treatment: durvalumab, dostarlimab, and pembrolizumab [68]. Dostalimab, branded as Jemperli, showed a mortality benefit in patients with MMRp and microsatellite stable (MSS) tumors, although smaller than in MMRd-MSI-H tumors [69]. Pembrolizumab, also known as Keytruda, plus paclitaxel and carboplatin, was studied in cohorts with MMRd and MMRp tumors. For advanced or recurrent EC specifically, the FDA approval was based on the NRG-GY018 phase III clinical trial. The results showed that patients with MMRd tumors in the pembrolizumab group had a 70% reduction in relative risk and an estimated PFS of 74% at the one-year mark, while the placebo group had 38% (HR = 0.30, 95% CI 0.19–0.48, *p* < 0.001) [70].

Surgical staging remains vital for determining therapy, and the majority of patients with EC achieve success with surgical intervention. However, individualized adjuvant treatment with chemotherapy, radiation, and/or novel immunotherapy continues to be an evolving research topic aimed at improving outcomes for these patients.

## 6. Conclusions and Prospects

EC can be a devastating disease, and significant advancements are currently being made to improve not only mortality but also PFS. In the early stages of EC, surgery with hysterectomy and BSO was the mainstay of treatment, with high success rates. Additional treatments with hormone therapies also provide fertility-sparing options for younger patients. EC is a complex disease with numerous variables contributing to its overall development and prognosis. Of particular importance to this paper are diabetes and obesity, which have both been identified as independent risk factors and prognostic factors, but less is established in the literature about how they interact in EC. Therefore, this review has sought to define that when patients have diabetes and obesity as comorbidities, not only is there an increase in the risk of EC development, but there are also worsened treatment outcomes and long-term survival. Diabetes and obesity synergistically exacerbate insulin resistance, chronic inflammation, and hormonal imbalances, increase IGF-1, free estrogen, and other pro-cancerous biomarkers, and cause immune dysregulation. This microenvironment is prone to cancer cell proliferation, especially EC. Given the substantial prevalence and rising incidence of diabetes, obesity, and EC in the United States, this highlights the need for targeted interventions and translational research with a focus on how these comorbidities uniquely impact the pathophysiologic processes of EC.

## Data Availability

No new data were created or analyzed in this study.

## Abbreviations

EC = Endometrial cancer; OR = Odds ratio; BMI = Body mass index; RR = Relative risk.
